# Adenoviral Vector Driven by a Minimal Rad51 Promoter Is Selective for p53-Deficient Tumor Cells

**DOI:** 10.1371/journal.pone.0028714

**Published:** 2011-12-09

**Authors:** Vincent Fong, Marika Osterbur, Cristina Capella, Yo-El Kim, Christopher Hine, Vera Gorbunova, Andrei Seluanov, Stephen Dewhurst

**Affiliations:** 1 Department of Microbiology and Immunology, University of Rochester, Rochester, New York, United States of America; 2 Department of Biology, University of Rochester, Rochester, New York, United States of America; Kantonal Hospital St. Gallen, Switzerland

## Abstract

**Background:**

The full length Rad51 promoter is highly active in cancer cells but not in normal cells. We therefore set out to assess whether we could confer this tumor-selectivity to an adenovirus vector.

**Methodology/Principal Findings:**

Expression of an adenovirally-vectored luciferase reporter gene from the Rad51 promoter was up to 50 fold higher in cancer cells than in normal cells. Further evaluations of a panel of truncated promoter mutants identified a 447 bp minimal core promoter element that retained the full tumor selectivity and transcriptional activity of the original promoter, in the context of an adenovirus vector. This core Rad51 promoter was highly active in cancer cells that lack functional p53, but less active in normal cells and in cancer cell lines with intact p53 function. Exogenous expression of p53 in a p53 null cell line strongly suppressed activity of the Rad51 core promoter, underscoring the selectivity of this promoter for p53-deficient cells. Follow-up experiments showed that the p53-dependent suppression of the Rad51 core promoter was mediated via an indirect, p300 coactivator dependent mechanism. Finally, transduction of target cells with an adenovirus vector encoding the thymidine kinase gene under transcriptional control of the Rad51 core promoter resulted in efficient killing of p53 defective cancer cells, but not of normal cells, upon addition of ganciclovir.

**Conclusions/Significance:**

Overall, these experiments demonstrated that a small core domain of the Rad51 promoter can be used to target selective transgene expression from adenoviral vectors to tumor cells lacking functional p53.

## Introduction

Specific targeting of therapeutic agents to cancer cells while avoiding damage to normal tissue has been a long time goal in cancer research. One method of targeting viral agents has been to use tumor specific promoters to restrict expression of therapeutic genes [1], [2]. Expression of the DNA repair gene, Rad51, has been shown to be upregulated in many cancers [3], [4], [5], especially higher grade [6], [7], [8], [9] chemoresistant [10] and radioresistant tumors [11]. The Rad51 protein plays a key role in homologous recombination [12]. Expression is tightly regulated in normal cells, with dysregulation leading to genomic instability and possibly contributing to oncogenesis [13], [14], [15], [16], [17].

Recently, Gorbunova and colleagues reported that the full length Rad51 promoter maintains its cancer specificity when taken independent of its natural context and showed that it can drive tumor-selective expression of a reporter gene [18]. This makes the Rad51 promoter a very attractive candidate for use in anti-cancer therapies especially when coupled with the efficient transduction capabilities of viral vectors [19]. We therefore conducted experiments to examine the feasibility of using the Rad51 promoter to drive tumor-selective expression of a transgene of interest from an adenovirus vector.

An essential initial objective was to define the minimal Rad51 promoter element that retained the robust transcriptional activity and tumor selectivity of the intact promoter, since the full length Rad51 promoter reported by Gorbunova and colleagues is over 6.5 kb in length [18] and exceeds the insert capacity for many adenoviral vectors [20], [21]. Our experiments succeeded in identifying a minimal core promoter element of approximately 450 bp that retained the full tumor selectivity and transcriptional activity of the intact promoter. We also found that the Rad51 promoter was more active in cancer cells that lacked functional p53, compared to cells with normal p53 (including both normal cells and cancer cells with intact p53 function). We then proceeded to evaluate the ability of this minimal core promoter to drive selective expression of the herpes simplex virus type 1 (HSV) thymidine kinase (TK) gene from an adenoviral vector in p53 defective cancer cells. Our studies showed ganciclovir dependent killing of transduced p53 defective cells with little effect on normal cells. These data suggest that the Rad51 core promoter may have utility in virally vectored gene therapies for p53 defective cancers.

## Results

### Determination of the Rad51 core promoter region

**Figure 1:**
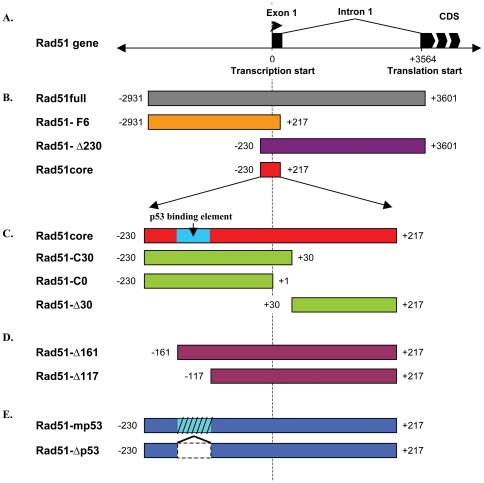
Rad51 promoter constructs. (A) Diagram of the Rad51 gene and upstream region. All labeled positions are specified relative to the transcription start site. (B) Diagram of the different Rad51 promoter truncations. (C) Diagram of the truncations of the Rad51 core region. A putative p53 binding region has been reported at position −161 to −117. (D) Diagram of promoter mutations affecting the p53-binding region.

Previous attempts to define the minimal Rad51 promoter have yielded conflicting results and were performed only in a single osteosarcoma cell line, U2-OS [22], [23]. In order to better assess the differential expression of the Rad51promoter, we generated a panel of truncated Rad51 promoter mutants (Figure 1), inserted them upstream of a promoterless luciferase reporter and produced a series of replication-defective, E1-deleted Ad5 vectors that were evaluated in a panel of normal and cancer cell lines (Table 1).

**Table 1:** Description of cell lines used in this study.

Cell Lines	Known p53 defect	Description	Citation
**Normal cell lines**			
Wi-38	None	Normal lung fibroblasts	[42]
HCA-2	None	Foreskin fibroblasts immortalized with hTERT	[43]
BJ	None	Foreskin fibroblasts	[45]
MRC-5	None	Normal lung fibroblasts	[47]
SAEC	None	Normal Small Airway Epithelial Cells (Clonetics)	N/A (Primary cell)
nHeps	None	Normal human primary hepatocytes (Clonetics)	N/A (Primary cell)
**Cancer cell lines**			
A549	None	Lung carcinoma	[46]
BxPc-3	Mutation Y220C	Pancreatic adenocarcinoma	[31]
MCF-7	None	Breast adenocarcinoma	[46]
H1299	p53null	Non-small cell lung carcinoma	[48]
HeLa	Likely degradation by HPV E6	Cervical adenocarcinoma	[44]
SF-539	None	Glioma	[46]
U251	Mutation R273H	Glioma	[46]

**Figure 2:**
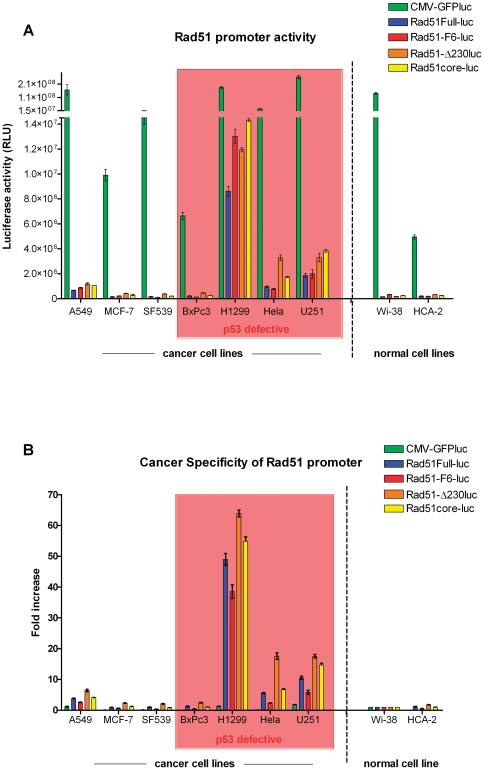
Identification of a core Rad51 promoter element. (A) Luciferase assays measuring Rad51 promoter activity in 9 different cancer and non-cancer cell lines. Cells were transduced with their respective vectors at a MOI of 100. 24 hours post-infection, cells were lysed and luciferase activity of the cell lysates measured. Data are presented as mean values of independent experimental triplicates; error bars represent the standard deviation of the data values. (B) As a measure of tumor specificity, luciferase activity for each construct in each cell type was calculated as a ratio relative to its activity in normal lung fibroblasts (Wi-38).

As can be seen in Figure 2, maximal promoter strength was retained by a small DNA region surrounding the transcription start site (−230/+217). Luciferase activity in cells transduced with a vector containing this element (Rad51core-luc) was essentially indistinguishable from that in cells transduced with vectors containing larger fragments of the Rad51 promoter (Rad51-F6-luc, −2931/+217; Rad51-Δ230-luc, −230/+3564) or the intact full-length Rad51 promoter (−2931/+3564) (Figure 2A). To assess the selectivity of the promoter elements for the various cell lines, the luciferase activities for each cell line were normalized to promoter activity in normal human lung fibroblasts, Wi-38 cells, which was arbitrarily assigned a value of 1. This analysis (Figure 2B) confirmed that maximal promoter activity and promoter selectivity were conferred by the Rad51 core promoter element (−230/+217). In contrast, the same analysis performed with a CMV promoter showed little to no tumor specificity.

In order to assess whether Rad51core promoter activity was correlated with endogenous cellular expression of Rad51 protein, nuclear extracts from each cell type were isolated and analyzed by Western blot for Rad51 protein (Information S1). A non-parametic statistical comparison of Rad51 protein content to Rad51core promoter activity in each cell line revealed a statistically significant correlation (Spearman rank correlation coefficient = 0.7, with a p-value = 0.04; Information S1).

**Figure 3:**
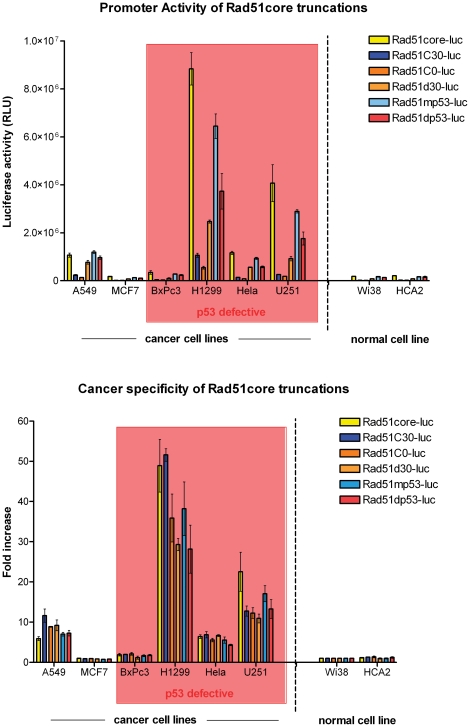
Further truncations of the Rad51 core promoter lead to progressive loss of transcriptional activity, while the reported p53 binding site is not required for tumor selectivity of the Rad51 core promoter. Truncations of the Rad51core promoter were created to determine if all parts were necessary (see Fig. 1C) and modifications to the Rad51core promoter were constructed to evaluate the contribution of putative p53 binding sites to promoter activity (see Fig. 1E). The p53 binding sites were either mutated at conserved sites (Rad51mp53) or deleted (Rad51dp53) (as shown in Fig. 1E). (A) Luciferase assays measuring Rad51 promoter activity in 8 different cancer and non-cancer cell lines. Cells were transduced with their respective vectors at a MOI of 100. 24 hours post-infection, cells were lysed and luciferase activity of the cell lysates measured. Data are presented as mean values of independent experimental triplicates; error bars represent the standard deviation of the data values. (B) As a measure of tumor specificity, luciferase activity for each construct in each cell type was calculated as a ratio relative to its activity in normal lung fibroblasts (Wi-38).

To test whether this core element could be refined further, we constructed a series of truncated derivatives of the Rad51core element (Figure 1C). Analysis of the transcriptional activity of these constructs revealed that all of them were significantly less active than the Rad51core element (Figure 3). We therefore focused subsequent studies on the Rad51core promoter construct.

### Rad51 promoter activity is suppressed by p53

**Figure 4:**
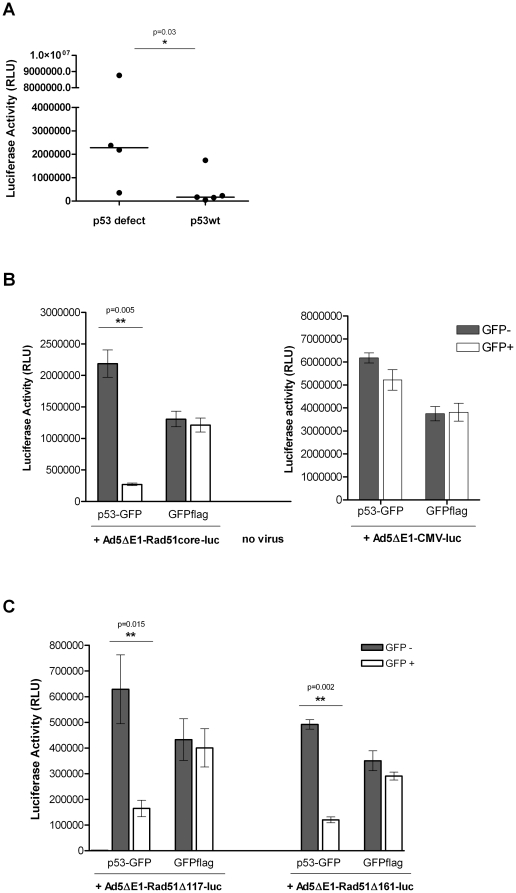
p53 suppresses Rad51 promoter expression. (A) Cells with functional p53 have lower Rad51 promoter activity. 9 different cancer and non-cancer cell lines were grouped by their reported p53 status and transduced with Ad5ΔE1-Rad51full-luc vector at a MOI of 100. 24 hours post-infection, cells were lysed and luciferase activity of the cell lysates measured. Each point represents the mean value of independent experimental triplicates of a cell line. The median value of each group, indicated by the line, was determined to be significantly different by a Mann-Whitney analysis (p = 0.03). (B) Restoring expression of p53 in a p53-/- cell line reduces the activity of the Rad51 core promoter. H1299 cells were transfected with a plasmid encoding either a p53-GFP fusion protein or GFP flag. 12 hrs post-transfection, the cells were transduced with Ad5ΔE1-Rad51core-luc vector or Ad5ΔE1-CMV-luc at a MOI of 100 pfu/cell. 24 hrs thereafter, the cells were collected and sorted into GFP+ and GFP− populations via FACS. The cells were then lysed and luciferase activity measured. Data are presented as mean values of independent experimental triplicates; error bars represent the standard deviation of the data value. The Rad51 promoter activity in p53 positive and negative cells was significantly different, as determined by a paired student's T-test (p = 0.005); in contrast, the CMV promoter activity was statistically equivalent in both p53 positive and negative cells. (C) P53 mediated suppression of Rad51 promoter activity was not dependent on a reported p53 binding region (−161/−117). Methods were as described in (B), except that cells were transduced with Ad5ΔE1-Rad51Δ161-luc vector or Ad5ΔE1-Rad51Δ117-luc vector instead of Ad5ΔE1-Rad51core-luc (see Fig. 1D for schematic representations of these vectors).

During the course of testing the Rad51 promoter truncations, we observed that the Rad51 promoter appeared to be more active in cells lacking functional p53, than in cells with intact p53 function. To formally test this hypothesis, we segregated our panel of 9 cell lines into lines previously reported to have defective or deleted p53, and those reported to have normal p53 function (Table 1). The median luciferase activity in cells transduced with Ad5ΔE1-Rad51full-luc was 272,000 RLU/20 µg cell lysate for cells with wild-type p53. In contrast, median promoter activity in cells lacking functional p53 was 10-fold higher, at 2,790,000 RLU/20 µg cell lysate (Figure 4A). Using a Mann-Whitney analysis we found that Rad51 promoter activity differed significantly between the 2 groups (p-value = 0.03; Figure 4A).

In order to confirm the inverse relationship between Rad51 promoter activity and p53 function, we transfected a p53-null cell line, H1299, with a plasmid encoding a p53-GFP fusion protein and then 12 hrs later transduced the same cells with Ad5ΔE1-Rad51core-luc or Ad5ΔE1-CMV-luc. After a further 24 hours, the cells were then flow cytometrically sorted into GFP+ and GFP− populations, lysed and luciferase activity was measured. When p53 function was restored to H1299 cells, Rad51core promoter activity was reduced to less than 15% of its original activity, while there was no significant change in CMV promoter activity (Figure 4B). This suggests that p53 has a strong and selective inhibitory effect on the Rad51core promoter. Additional experiments overexpressing p53 in HeLa cells yielded similar results - supporting that this inhibitory effect is not limited to H1299 cells or p53 null cells (Information S1).

### The p53 binding site is not required for tumor-selectivity of the Rad51 core promoter

It has been reported that the Rad51core promoter contains p53 binding elements within the region −159/−118 upstream of the transcription start [24]. To test the effect of this region on transcriptional activity of Rad51core, we derived a series of plasmid constructs, including (i) promoter truncations spanning this element (Figure 1D), and (ii) site-directed mutations or internal deletions of the binding sites (Figure 1E). We then inserted these constructs upstream of the promoterless luciferase reporter gene in our replication-defective Ad5 vector, and tested their transcriptional activity.

We first tested promoter constructs that were truncated either immediately upstream of the putative p53 binding domain (Rad51-Δ161, which contains the p53 binding region) or immediately downstream of this element (Rad51-Δ117, which lacks the p53 binding region) (Figure 1D). These constructs were introduced into p53-null H1299 cells that were then transfected with the p53-GFP expression plasmid described above, and FACS-sorted into GFP+ (p53+) and GFP− (p53−) populations. The transcriptional activity of both of these Rad51 promoter elements was suppressed in p53-positive cells (GFP+ in Figure 4C) not in p53-negative cells (GFP− in Figure 4C). Thus, the presence or absence of the p53 binding region within the Rad51 promoter (−161/−117) had no effect the ability of p53 to inhibit Rad51-mediated transcription.

We next examined the transcriptional activity of site-directed mutants of the Rad51 promoter, targeting the p53 binding region. The Rad51mp53 construct is identical to the Rad51core promoter, except for mutations to the conserved bases of the putative p53 binding sites, while the Rad51Δp53 construct contains a precise deletion of the p53 binding region (−159/−118) (Figure 1E). Each of these constructs was introduced into a panel of normal and cancer cell lines, along with the Rad51core promoter construct. The results (Figure 3) showed that the transcriptional activity of the Rad51 core element was unaffected either by mutation (Rad51mp53) or deletion (Rad51dp53) of the p53 binding region even in cells that expressed functional p53. Based on these data, and the results presented in Figure 4C, we conclude that p53 suppresses the transcriptional activity of the Rad51 promoter through a mechanism that is independent of the reported p53 binding site [24] within the Rad51 promoter.

### Overexpression of p300 relieves p53 mediated repression of the Rad51core promoter

**Figure 5:**
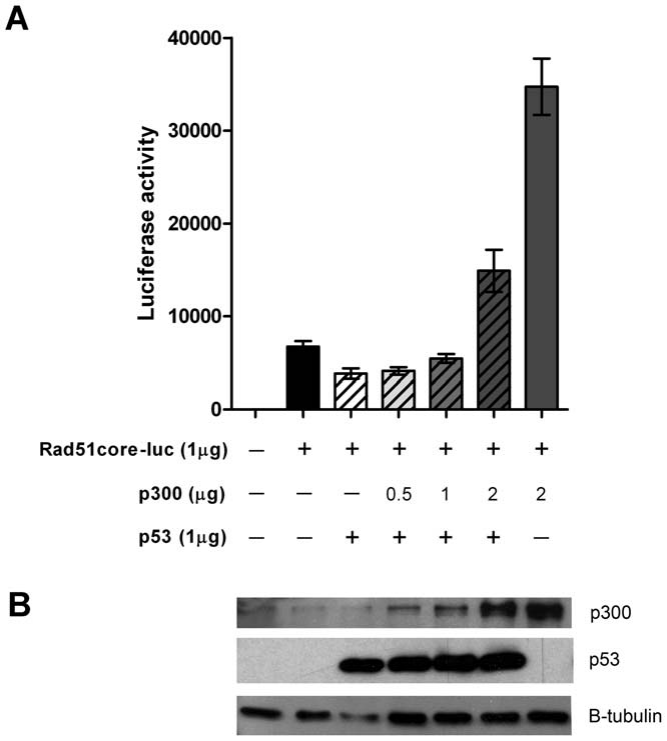
Overexpression of p300/CBP overcomes p53-mediated repression of Rad51 core promoter activity. H1299 cells were cotransfected with a plasmid expressing luciferase under the control of the Rad51core promoter, in the presence or absence of plasmids encoding wild-type p53 or p300. Increasing amounts of the p300 expression were used (as indicated); the total amount of amount of transfected plasmid DNA in each reaction was maintained at a constant 4 µg by adding the necessary amount of irrelevant pCMV-GFPflag plasmid DNA. (A) 48 hours post transfection, cells were collected, lysed and transcriptional activity measured by Luciferase assay. Results shown represent the mean of independent experimental triplicates; error bars represent the standard deviation of the data. (B) Western blot analysis was performed to confirm expression of p53 and p300. 10 µg of total protein from each sample was separated on a 7.5% polyacrylamide gel, transferred to a nitrocellulose membrane, and probed for each respective protein using antibodies specific for the indicated proteins (p300, p53 and β-tubulin as a loading control).

p300 is a ubiquitous coactivator of transcription that has been reported to interact with many different transcription factors [25], including p53 [26]. We therefore hypothesized that p53 might indirectly affect Rad51 promoter activity through interactions with p300. To test this prediction, we transfected p53null H1299 cells with the pShuttle-Rad51core-luc plasmid along with increasing amounts of p300 expressing plasmid in the presence or absence of a plasmid expressing wild-type p53. The GFPflag expressing plasmid, pCMV-GFPflag, was used to as “filler” to ensure equivalent quantities of DNA were transfected into each sample. Successful transfection and expression of p53 and p300 was confirmed by western blot (Figure 5B). Consistent with the data presented in Figure 4, expression of p53wt repressed transcriptional activity from the Rad51core promoter. However, overexpression of p300 relieved this p53 induced repression, in a dose dependent manner (Figure 5A). Moreover, when p300 was overexpressed at very high levels, Rad51core promoter activity was increased above basal levels (e.g., cotransfection of 2.0 µg of a p300 encoding plasmid increased transcriptional activity from the Rad51core promoter by 5.2-fold; Figure 5A).

### Tumor-selective induction of cell death, directed by the Rad51core promoter

**Figure 6:**
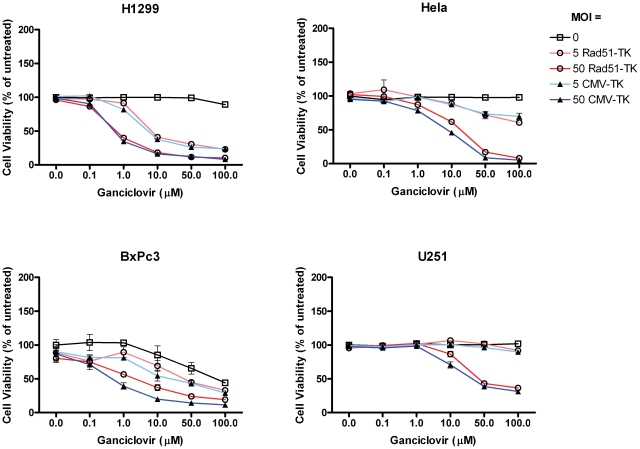
The Rad51core promoter can be used to selectively express a toxic gene in p53-defective cells. P53 defective cancer cells (as indicated) were transduced with a replication-defective Ad5 vector encoding the HSV thymidine kinase (TK) gene under the transcriptional control of the Rad51core element (Ad5ΔE1-Rad51core-TK) or the constitutively active CMV promoter (Ad5ΔE1-CMV-TK). Cells were transduced with the vector at the indicated MOIs (5 or 50), in the presence or absence of varying concentrations of ganciclovir (0–100 µM). Five days later, cell viability was determined with AlamarBlue™ dye and normalized as a percentage of the mean value of untreated cells. Data are presented as mean values of independent experimental triplicates; error bars represent the standard deviation of the data.

**Figure 7:**
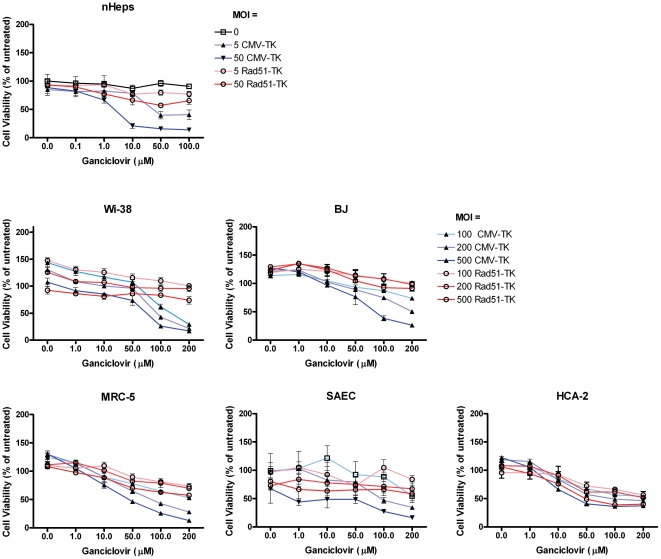
Expression of a toxic gene from the Rad51core promoter causes little toxicity in primary cells. Primary cells (as indicated) were transduced with high doses of a replication-defective Ad5 vector encoding the HSV TK gene under the transcriptional control of the Rad51core element (Ad5ΔE1-Rad51core-TK) or the constitutively active CMV promoter (Ad5ΔE1-CMV-TK). Cells were transduced with the vector at the indicated MOIs (5–500), in the presence or absence of varying concentrations of ganciclovir (0–200 µM). Five days later, cell viability was determined with AlamarBlue™ dye and normalized as a percentage of the mean value of untreated cells. Data are presented as mean values of independent experimental triplicates; error bars represent the standard deviation of the data.

Having defined the core Rad51 promoter, and demonstrated its regulation by p53, we proceeded to examine whether this DNA element could be used to selectively drive expression of a cytotoxic effector protein in p53-defective tumor cells. We constructed an adenoviral vector encoding HSV thymidine kinase under the control of the Rad51core promoter (Ad5ΔE1-Rad51core-TK). A panel of p53 defective cancer cells and non-cancer cell lines were transduced with Ad5ΔE1-Rad51core-TK and an Ad5ΔE1-CMV-TK control vector at various moi (0 to 500 pfu/cell). Cells were simultaneously treated with ganciclovir (GCV) at concentrations ranging from 0 to 200 µM. The p53-defective cancer cells clearly demonstrated a GCV dose dependent loss of viability with both vectors (Figure 6), while the primary cell lines only showed loss of viability in cultures transduced with the control, CMV-driven, vector (Figure 7). This is exemplified by the fact that, at an MOI of 50 pfu/cell, Ad5ΔE1-Rad51core-TK caused almost 60% loss of viability in H1299 cells (Figure 6) treated with 1 µM GCV, while treatment of BJ fibroblasts (Figure 7) with 10× more vector and 200× higher concentration of GCV resulted in less than 10% loss of viability. At these same doses, the control vector (Ad5ΔE1-CMV-TK) elicited in a similar ∼60% loss of viability in H1299 cells, but a much greater, ∼74% loss of viability, in BJ fibroblasts.

## Discussion

Two previous attempts to map the minimal Rad51 promoter, both performed in the U2-OS osteosarcoma cell line, identified different essential promoter domains: −204/−58 [22] and −536/−412 [23]. In this report, we performed a more comprehensive analysis of the Rad51 promoter in a broad range of cell lines, including both cancer cells and primary cells. Our experiments defined a small (447 bp) element from the Rad51 promoter that supports cancer specific transcriptional activity, and is sufficiently compact to allow its use in DNA delivery systems where transgene capacity is limited. We found no evidence for the presence of strong transcriptional suppressor or enhancer elements flanking this core element [22].

The broad range of cancers in which the core Rad51 promoter is active may be explained by its increased activity in cells lacking the tumor suppressor p53, one of the most commonly mutated genes in cancer [27]. Our data show that p53 suppresses the activity of the Rad51 promoter, and that Rad51 promoter activity in different cell lines has an inverse relationship to p53 function. Two exceptions to this trend were noted: A549 cells (where the Rad51 promoter was highly active) and BxPc-3 cells (where activity was lower than in other p53-defective cells). We tentatively attribute this to additional regulatory dysfunction in these cancer lines. A549 cells have been reported to have an overexpression of MDM-2 [28], which promotes nuclear export [29] and degradation of p53 protein [30]. As a consequence, Lu et al. were unable to detect p53 in A549 cells by immunocytochemical staining, and they reported low levels of p53 in the cell even after transduction with exogenous p53 DNA. This suggests that, while the p53 gene is normal in A549 cells, protein expression levels are extremely low – and possibly too low to effectively suppress Rad51-driven gene expression. In the case of the BxPc-3 pancreatic cancer cell line, we confirmed that the previously described mutation at codon 220 of the p53 gene in this cell line was indeed present [31]. However, BxPc-3 cells have been shown to express high levels of mutant p53 [3], [31]. Thus, one possible explanation for our data is that the overabundance of this mutant isoform of p53 in BxPc-3 cells actually serves to further sequester p300 (resulting in low levels of Rad51-driven luciferase expression). Additionally, pancreatic cancer cells have been shown to accurately reflect their malignant phenotype [3], in which Rad51 is overexpressed, only when cultured in a three dimensional matrix.

Overall, our results are broadly consistent with a previous report that p53 can suppress the transcriptional activity of the Rad51 promoter [24]. Contrary to this study, however, we found that p53-mediated suppression of Rad51 promoter activity occurred independently of the reported p53 binding region in the promoter [24]. This may reflect methodological differences, since we studied the core Rad51 promoter (−230/+217) in the context of a linear adenoviral vector, whereas Arias-Lopez et al. studied a larger fragment of the promoter (−948/+1427) in the context of a circular plasmid vector [24]. Regardless, our data strongly suggest that p53 can act an indirect manner, possibly through effects on other transcription factors or cofactors that may regulate the Rad51 promoter.

p300 is a well known co-activator of transcription, shown to interact with p53 and many other transcription factors [25]. Here we showed that overexpression of p300 increased transcriptional activity from the Rad51core promoter and relieved the p53 mediated repression (Figure 5). This suggests that p53 may have sequestered limiting amounts of p300 that otherwise could have activated Rad51 promoter activity. The fact that p300 overexpression increased Rad51core promoter activity even in the absence of p53 further supports the idea that p300 is limiting with respect to the transcriptional activity of the Rad51core promoter, and suggests that some additional inhibitory factors may be acting on this promoter in a p300-dependent manner.

It has been previously suggested that competition by transcription factors for binding to limiting amounts of p300/CBP protein may lead to transcriptional repression of one of the competing gene targets [32], [33]. Furthermore p53 has been shown to transcriptionally repress target genes through interaction with p300/CBP binding partners, such as Ets1/2 [34], Sp1 [35] and C/EBP [36]. Analysis of the Rad51core promoter using the Transcription Element Search System (TESS) [37], revealed potential binding sites for the transcription factors Sp1, C/EBP, and E2F-1. Hasselbach et al. also reported binding sites and DNA binding by E2F-1 as well as by STAT-5 within the Rad51 promoter [22], and Rad51 expression has been shown to be regulated by E2F-1 [38] and STAT5 [39]. Since all of these transcription factors can be activated through interactions with p300/CBP [25], [40], competitive binding of p53 to p300/CBP offers a likely mechanism through which p53 may repress the Rad51 core promoter. Alternatively Rad51 may be also regulated by the many changes to cellular metabolism that occur during oncogenic transformation, some of which may be the result of a p53 defect.

Finally, we demonstrated the ability of a Rad51core driven adenoviral vector to achieve selective cell killing of p53-defective cancer cells (Figure 6), while sparing normal cells (Figure 7). The Ad5ΔE1-Rad51core-TK and Ad5ΔE1-CMV-TK vectors were equally efficient in mediating ganciclovir-dependent killing of p53 defective cancer cells. This demonstrates the robust ability of the Rad51 promoter to drive expression of a cytotoxic transgene in cancer cells. Moreover the Ad5ΔE1-Rad51core-TK vector was not cytotoxic to normal cell lines, whereas the Ad5ΔE1-CMV-TK vector elicited GCV-dependent cell death even in normal cells. The toxicity of the CMV-driven vector demonstrates that adenoviral vectors can efficiently transduce normal cells, underscoring the selectivity and potential utility of the Rad51 promoter. The efficacy with which the vector killed p53-defective tumor cells was correlated with the transcriptional activity of the Rad51core promoter in the various cells. Overall, these data establish proof of principle support for the use of the Rad51core promoter in virally vectored gene therapies for p53 defective cancers.

## Materials and Methods

### Cell culture

HEK 293A [41], Wi-38 [42], HCA-2 [43], Hela [44], BJ [45], MCF-7 [46], A549 [46] and MRC-5 [47] cells were cultured in Dulbecco's Modified Eagle Medium (Gibco) supplemented with 10% FBS (Gibco) and 1× Pen Strep Glutamine (Gibco). H1299 [48], BxPc-3 [31], U251 [46], SF-539 [46] cells were cultured in RPMI 1640 medium (Gibco) supplemented with 10% FBS (Gibco) and 1× Pen Strep Glutamine (Gibco). SAEC (Lonza) and nHeps (Lonza) cells were cultured in the growth media suggested by the vendor.

### Cloning of Rad51 promoters

The Rad51full promoter was PCR amplified from human genomic DNA and cloned into peGFP (Clontech) as described [18]. All subsequent truncations were made by PCR or restriction enzyme digestion from peGFP-Rad51full. Rad51-mp53 and Rad51-Δp53 were commercially synthesized by GeneArt (Regensburg, Germany). Rad51-mp53 is derived from Rad51core and has the underlined nucleotides in the region −159/−118 mutated from AAACTCGCGCAGGATCAAGCTCTCGAGCTCCCGTCTTGGGT to AAA**GG**C**C**CGCAGGAT**GGCC**CTCTCGAGCTCCCGT**GGCC**GGT. Rad51-Δp53 is also derived from Rad51core but has the entire region deleted.

### Virus construction

All adenovirus vectors were made with the AdEasy™ system (Stratagene). Rad51 promoter constructs were cloned upstream of a luciferase cassette in a pShuttle backbone, recombined with pAdEasy and linearized to form an adenoviral genome. This DNA was transfected into HEK 293A cells for virus production and amplification. Viruses were collected, purified on a CsCl gradient and titered by plaque assay. A control vector expressing luciferase and GFP from the constitutive CMV promoter, Ad5ΔE1-CMV-GFPluc, was created by cloning the luciferase gene from pGL3-basic into the pAdtrack shuttle plasmid and recombined into pAdeasy; virus production was then completed as described above. A control vector that expressed only luciferase under the control of the CMV promoter, Ad5ΔE1-CMV-luc, was purchased from Vector Biolabs (Philadelphia, PA).

### Measuring promoter activity

Each cell type was seeded at a density of 5×10^5^ cells per well into 6-well plates and cultured overnight. The following morning, the cell culture media was removed and replaced with fresh media containing the indicated adenoviral vectors at a multiplicity of infection (MOI) of 100 pfu/cell. 24 hours after transduction the cells were collected and lysed with Passive Lysis Buffer (Promega). Luciferase assays were performed with Luciferase Assay System from Promega and luminescence measured on a DTX880 multimode plate reader (Beckman Coulter). All samples were normalized by total protein content as determined by Bradford assay.

### p53 rescue experiment

1×10^6^ H1299 cells were seeded in 30 mm cell culture plates and cultured overnight. The following morning, 4.0 µg of either pEGFP-p53 or pCMV-GFPflag was transfected into the cells using Lipofectamine2000 (Invitrogen). 12 hrs post-transfection, the cells were infected with Ad5ΔE1-Rad51core-luc or Ad5ΔE1-CMV-luc at an MOI of 100. 24 hrs post-infection, the cells were collected and sorted into GFP+ and GFP− populations on a FACSAriaII cell sorter. The cells were then lysed and luciferase activity measured with the Luciferase Assay System from Promega, described above. The experiment presented in Information S1, using HeLa cells, was performed using essentially methods.

### Co-activator overexpression experiments

H1299 cells were seeded in 6-well plates at a density of 2.5×10^5^ cells/well. The following day cells were cotransfected with a plasmid expressing luciferase under the control of Rad51core promoter (1 µg pShuttle-Rad51core-luc), in the presence or absence of plasmids expressing p53wt (1 µg pCMV-p53wt), p300 (0.5–2 µg pCMV-p300) and GFPflag (pCMV-GFPflag) using Lipofectamine2000 (Invitrogen). The amount of irrelevant GFPflag plasmid (“filler”) DNA was adjusted to maintain a total of 4 µg transfected DNA in all conditions. 6 hours post transfection, the serum free transfection medium was replaced with fresh culture medium and cell were then incubated for 48 hours at 37° prior to collection, lysis and performance of luciferase assays, as described above.

### GCV/HSVtk treatment and cell viability measurement

Cells were seeded in 96-well plates at a density of 1×10^3^ cells per well. 24 hours later, culture media was aspirated and replaced with fresh media containing appropriate dilutions of ganciclovir and vector. The cells were returned to incubate at 37° C for 5 days after which cells were stained with AlamarBlue™ (Invitrogen). Cell culture media was removed from cells, replaced with fresh media + AlamarBlue™, and cells were then incubated at 37°C for an additional 12 hours. Fluorescence (EX = 535 nm, EM = 595 nm) was measured in a DTX880 multimode plate reader (Beckman Coulter). Cell viability was calculated as a percentage of the mean value of untreated cells.

## Supporting Information

Information S1 **Rad51core promoter activity correlates with host cell expression of Rad51 protein.** (A) Nuclear extracts were isolated from each cell type and 3 µg of total protein was separated on a 10% polyacrylamide gel, transferred to a nitrocellulose membrane, and probed with a monoclonal antibody for Rad51. The blots were stripped and re-probed for β-tubulin as a loading control. All displayed samples were run on the same blot. (B) Rad51 expression was quantitated by densitometry from the blot shown in panel (A) using QuantityOne software (BioRad); Rad51 expression was then normalized in terms of housekeeping protein levels (β-tubulin). Rad51 expression levels in normal fibroblasts (Wi-38 cells) were defined as 1, and Rad51 expression levels in the other cells were then expressed relative to this. Shown is a scatterplot of endogenous Rad51 protein content for each cell line, versus the level of Rad51core promoter activity in the same cell line. Statistical analysis was performed using a non-parametric test and determined to be significant (Spearman rank correlation coefficient = 0.7, p = 0.04).
(PDF)
