# Supplementary material for: Adenoviral Vector Driven by a Minimal Rad51 Promoter Is Selective for p53-Deficient Tumor Cells
Source: PLoS One. 2011 Dec 9;6(12):e28714. doi: 10.1371/journal.pone.0028714 (PMC3235156; doi:10.1371/journal.pone.0028714)
Supplement: Information S1 — Rad51core promoter activity correlates with host cell expression of Rad51 protein. (A) Nuclear extracts were isolated from each cell type and 3 µg of total protein was separated on a 10% polyacrylamide gel, transferred to a nitrocellulose membrane, and probed with a monoclonal antibody for Rad51. The blots were stripped and re-probed for β-tubulin as a loading control. All displayed samples were run on the same blot. (B) Rad51 expression was quantitated by densitometry from the blot shown in panel (A) using QuantityOne software (BioRad); Rad51 expression was then normalized in terms of housekeeping protein levels (β-tubulin). Rad51 expression levels in normal fibroblasts (Wi-38 cells) were defined as 1, and Rad51 expression levels in the other cells were then expressed relative to this. Shown is a scatterplot of endogenous Rad51 protein content for each cell line, versus the level of Rad51core promoter activity in the same cell line. Statistical analysis was performed using a non-parametric test and determined to be significant (Spearman rank correlation coefficient = 0.7, p = 0.04). (PDF) [file pone.0028714.s001.pdf]

SUPPORTING INFORMATION S1

Rad51core promoter activity correlates with host cell expression of Rad51 protein.

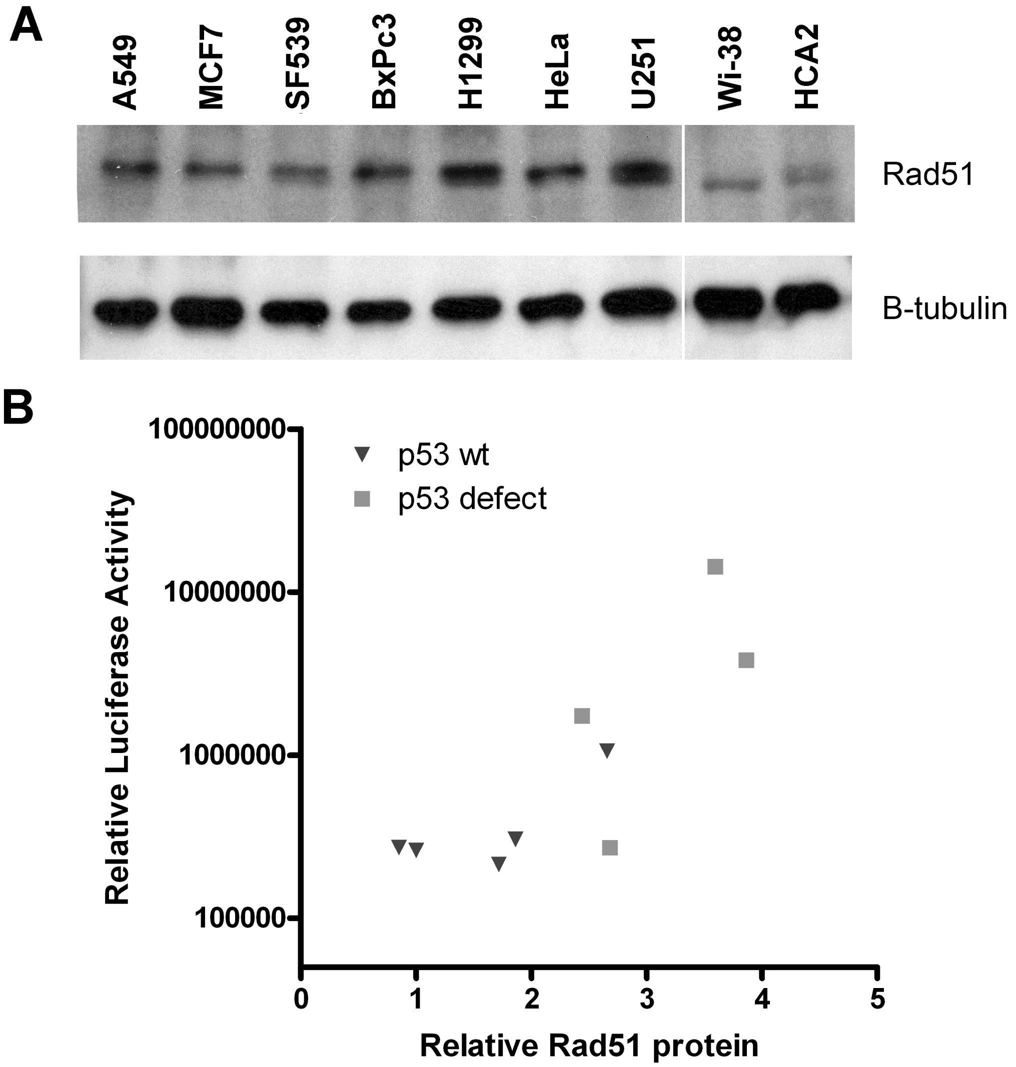

LEGEND. (A) Nuclear extracts were isolated from each cell type and 3μg of total protein was separated on a 10% polyacrylamide gel, transferred to a nitrocellulose membrane, and probed with a monoclonal antibody for Rad51. The blots were stripped and re-probed for β-tubulin as a loading control. All displayed samples were run on the same blot. (B) Rad51 expression was quantitated by densitometry from the blot shown in panel (A) using QuantityOne software (BioRad); Rad51 expression was then normalized in terms of housekeeping protein levels (β-tubulin). Rad51 expression levels in normal fibroblasts (Wi-38 cells) were defined as 1, and Rad51 expression levels in the other cells were then expressed relative to this. Shown is a scatterplot of endogenous Rad51 protein content for each cell line, versus the level of Rad51core promoter activity in the same cell line. Statistical analysis was performed using a non-parametric test and determined to be significant (Spearman rank correlation coefficient = 0.7, p = 0.04).

Overexpression of p53 in HeLa cells suppresses the transcriptional activity of the Rad51 core promoter

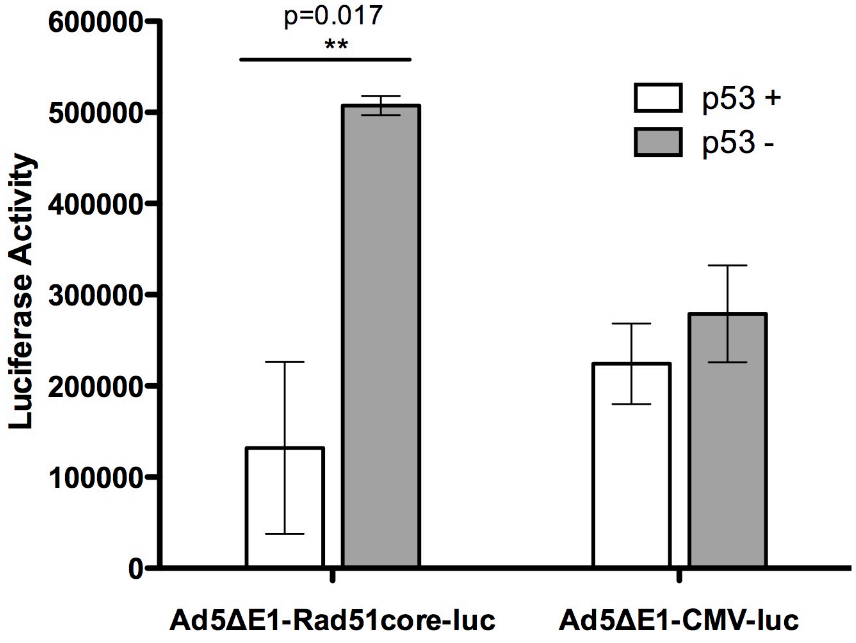

LEGEND. HeLa cells were transfected with a plasmid encoding a p53-GFP fusion protein. 12 hrs post-transfection, the cells were transduced with Ad5ΔE1-Rad51core-luc vector or Ad5ΔE1-CMV-luc at a MOI of 100 pfu/cell. 24 hrs thereafter, the cells were collected and sorted into GFP+ and GFP- populations via FACS. The cells were then lysed and luciferase activity measured. Data are presented as mean values of independent experimental triplicates; error bars represent the standard deviation of the data value. The Rad51 promoter activity in p53 positive and negative cells was significantly different, as determined by a paired student's T-test ($p = 0.017$); in contrast, the CMV promoter activity was statistically equivalent in both p53 positive and negative cells.
